# Pet Ownership and Multiple Sclerosis during COVID-19

**DOI:** 10.3390/ijerph182312683

**Published:** 2021-12-01

**Authors:** Holly Oliver-Hall, Elena Ratschen, Christopher R. Tench, Helen Brooks, Cris S. Constantinescu, Laura Edwards

**Affiliations:** 1 Division of Medical Sciences and Graduate Entry Medicine, Royal Derby Hospital, University of Nottingham, Uttoxeter Road, Derby DE22 3DT, UK; hollyoliverhall@aol.com; 2 Department of Health Sciences, University of York, York YO10 5DD, UK; Elena.ratschen@york.ac.uk; 3 Mental Health & Clinical Neurosciences, Clinical Neurology, University of Nottingham, Queen’s Medical Centre, Nottingham NG7 2UH, UK; mszct@nottingham.ac.uk (C.R.T.); cris.constantinescu@nottingham.ac.uk (C.S.C.); 4 NIHR Nottingham Biomedical Research Centre, Queen’s Medical Centre, University of Nottingham, Nottingham NG7 2UH, UK; 5 Division of Nursing, Midwifery and Social Work, School of Health Sciences, Manchester Academic Health Science Centre, University of Manchester, Oxford Road, Manchester M13 9PL, UK; helen.brooks@manchester.ac.uk; 6 Department of Neurology, Cooper University Hospital, Camden, NJ 08103, USA; 7 Division of Rehabilitation Medicine, Florence Nightingale Community Hospital, University Hospitals of Derby and Burton NHS Foundation Trust, London Road, Derby DE1 2QY, UK

**Keywords:** multiple sclerosis, pet ownership, COVID-19, quality of life

## Abstract

**Background:** Multiple sclerosis (MS) is associated with lower quality of life, reduced social participation, and decreased self-efficacy. The COVID-19 pandemic has had documented effects on the health and wellbeing of people with and without MS. Previous research has demonstrated the positive impact pets can have for people living with long-term conditions. **Objectives:** To explore the rates of pet ownership and pet attachment in people living with MS and pet ownership associations with quality of life, satisfaction with social roles, and self-efficacy scores; and to explore the effects of the COVID-19 outbreak on people’s perceived relationships with their pets. **Materials and Methods:** A postal questionnaire was distributed to members of a local MS Register and a control group of people without MS. The questionnaire assessed quality of life, satisfaction with social roles, self-efficacy, the perceived roles of pets, and pet-related concerns experienced during the COVID-19 pandemic. **Results:** No apparent difference in attachment to pets was found between the patient and control groups. Pet ownership and level of attachment were not associated with differences in quality of life or self-efficacy scores in people living with MS. Using multiple regression analysis, pet ownership was associated with a decrease in satisfaction with participation in social roles, but with the estimated effect being small compared to having a diagnosis of MS or being unemployed. Most participants reported that pets had positive roles during the pandemic, and the most reported pet-related concern was access to veterinary treatment. **Conclusion:** Pet owners both with and without MS reported subjective benefits to their wellbeing from pet ownership during COVID-19, although analysis suggested that pet ownership was associated with a reduction in satisfaction with social roles. The study had several limitations and suggestions are made for future work.

## 1. Introduction

Multiple sclerosis (MS) is a demyelinating disease that affects the central nervous system. It can cause a wide range of signs and symptoms, with motor, cognitive, and emotional effects among others. Fatigue, depression, impaired bladder and bowel control, muscle weakness and stiffness, cognitive impairment, and pain are common [1,2,3,4] and it is perhaps unsurprising that people with MS (PwMS) have reduced quality of life [5,6], mood [7], and social participation [8] compared to people without MS.

There is some evidence that pet ownership can benefit health and wellbeing across a variety of conditions [9,10], increasing life satisfaction, engendering a sense of responsibility and routine, and providing unconditional love, companionship, social interaction, and physical contact [11,12,13]. Individuals with chronic physical and/or mental health conditions often report that their pets improve their quality of life and help with symptom management [14,15].

Self-efficacy is the belief in one’s ability to succeed in specific situations or accomplish tasks [16]. It has been identified as a key factor in people being able to live successfully with the multiple challenges of chronic illnesses [17]. In MS, enhancing self-efficacy has been shown to improve health-related quality of life and reduce anxiety and depression [18,19]. Previous work in health and in chronic illness supports the notion that pet ownership improves self-efficacy [13,20]. 

Social roles are a way of defining how individuals behave and function in different groups, at work, at home, and at leisure. People participate in society at many different levels, as workers, family members, colleagues, friends, etc. People living with chronic illness often experience physical, psychological, and social barriers to full participation in society and societal roles [21], and may find it harder to relate to individuals, groups, and the overall community. Satisfaction with social roles has been shown to be reduced in PwMS [22]. We hypothesized that, particularly in individuals who may be more socially isolated, the companionship and “job of work” associated with caring for a pet may generate more satisfaction with social roles. In some chronic conditions, satisfaction with social roles is thought to mediate the relationships between symptoms or functioning and emotional outcomes [23].

Pet attachment can provide a form of social support and has been identified as being important in some people living with chronic illness [11], lessening the impact of social isolation [24]. 

The potential psychosocial benefits of pet ownership have not yet been explored in MS.

The COVID-19 pandemic has led to major changes in social interaction and concerns about risks of infection, particularly in those with underlying health conditions. Throughout the pandemic, higher than normal levels of both depression and anxiety have been recorded in the UK population [25]. PwMS have experienced a greater burden of depressive symptoms, worse sleep quality, and perceived increases in levels of fatigue than the general population [26,27,28], potentially exacerbating existing problems and further increasing inequalities in the health status between PwMS and those without MS.

Recent research suggests that pet ownership may have helped many people to cope during the COVID-19 pandemic. An online survey of pet owners reported that over 80% of respondents felt that their pets helped them to cope emotionally with COVID-19, and over 90% of participants said that they could not imagine being without their pet at this time [29]. Despite this, over 50% of people surveyed experienced pet-related worries, including accessibility to and affordability of veterinary care [29,30]. Animal ownership was associated with smaller decreases in mental health and smaller increases in loneliness during the pandemic [29]

### 1.1. Objectives

1. To explore the rates of pet ownership and associations with quality of life, satisfaction with social roles, and self-efficacy scores in PwMS.
2. To compare the levels of attachment to pets in PwMS and people without MS.
3. To compare the levels of satisfaction with social roles in pet-owning and non-pet-owning PwMS and people without MS.
4. To explore the effects of the COVID-19 outbreak on people’s perceived relationships with their pets (how the participants thought their pets helped them emotionally and physically and whether they had any concerns about being a pet owner during the COVID-19 pandemic).

### 1.2. Hypotheses:

1. Pet ownership is associated with higher rates of quality of life, self-efficacy, and satisfaction with social roles in PwMS.
2. Pet attachment may be higher in PwMS than in controls.
3. Satisfaction with social roles may be higher in pet-owning than non-pet-owning PwMS and controls.
4. Objective 4 is exploratory; no specific hypothesis generated.

## 2. Materials and Methods

The study received ethical approval from Social Care Research Ethics Committee (IRAS ID 261959) and the sponsor was the University of Nottingham (ref 19070).

### 2.1. Study Design

This was a cross-sectional questionnaire study involving PwMS as cases and controls (people without a diagnosis of MS). Cases and controls were subsequently further subdivided by pet ownership status, into pet owners and non-pet owners. 

### 2.2. Population

*PwMS/Cases/Patients*: Patients with a confirmed diagnosis of MS were recruited from the MS Register maintained at Nottingham University Hospitals NHS Trust. A questionnaire pack (containing invitation letter, information letter, questionnaire, and stamped addressed envelope as well as 2 “control packs” containing the same material) was posted to 800 individuals on this register. Patients receiving the questionnaire were asked if they would be willing to pass on one or two questionnaires to “controls” (defined as friends or non-blood relatives of the person with MS). 

Inclusion criteria:

- Aged 18 years or over.
- Diagnosis of MS AND on the Nottingham MS Register.

Exclusion criteria:

- Inability or unwillingness to complete the questionnaire.

*Controls*: Recruited patients were given the option of recruiting one or two friends or non-blood relatives without MS to whom they could pass on one of the “control packs”.

Inclusion criteria:

- Aged 18 years or over.
- Friend or non-blood relative of person with MS diagnosis.
- Not diagnosed with MS.

Exclusion criteria:

- Inability or unwillingness to complete questionnaire.

### 2.3. Questionnaire

The questionnaire comprised multiple-choice and short-answer questions including some validated questionnaires.

### 2.4. Demographic Data

Demographic data collected included current age, gender, employment status, and MS subtype (primary progressive (PP)MS/progressive relapsing (PR)MS/relapsing remitting (RR)MS/secondary progressive (SP)MS/Other/Not sure).

Data were not collected about co-morbidities.

### 2.5. Pet Ownership

Participants were asked to answer ‘yes’ or ‘no’ in response to the question, ‘do you own any pets?’. If participants answered ‘yes’, they were asked to record the species and number of pets owned. Pet owners and non-pet owners were invited to complete all parts of the questionnaire apart from those questions relating specifically to relationships with pets (e.g., the Lexington attachment to pets scale; perceived roles of pets during the pandemic).

There was no restriction by type or number of pets. 

**Attachment to pets:** Attachment to pets was assessed using the validated Lexington attachment to pets scale (LAPS), a 23-item questionnaire scored using a Likert scale with excellent psychometric properties [31]. Negative statements were scored in reverse and individual question scores were added to generate a total score. Higher scores indicate greater attachment.

**Satisfaction with (participation in) social roles (SPSR):** This was measured using the Patient-Reported Outcomes Measurement Information System (PROMIS) 8-item short form with a 5-point Likert scale, with the raw score being converted into a standardized “T” score, with higher scores indicating greater satisfaction with social roles. This is suitable for use in a postal questionnaire, being reliable, quick, and easy to complete [32,33].

**Quality of life (QoL):** QoL was assessed using the Leeds Multiple Sclerosis Quality of Life scale (LMS-QoL), an eight-item measure of QoL designed specifically for use by PwMS [34]. The instrument is brief, easy to complete, and provides a valid measure of wellbeing-associated QoL in PwMS [34,35,36]. Higher scores indicate a lower quality of life.

**Self-efficacy:** This was assessed using the 12-item Unidimensional Self-Efficacy scale for Multiple Sclerosis (USE-MS) [37]. It is both a reliable and valid means of assessing self-efficacy in PwMS [37]. Higher scores indicate increased self-efficacy.

**Perceived roles of pets during the pandemic:** Participants were asked to indicate their level of agreement with a series of five statements regarding the roles of their pets during the pandemic using a Likert scale with the following answer options: ‘strongly agree’, ‘somewhat agree’, ‘disagree’, ‘strongly disagree’. The statements included concerned the effects of pets on family (having positive effects or causing problems), physical activity (“keeping fit and active”), social interaction (“my animal is the reason I keep in touch with some people and social media groups”), and emotional coping (“my animal helps me cope emotionally with the COVID-19 situation”) during the pandemic. Participants were also asked to answer ‘yes’, ‘no’, or ‘prefer not to say’ to indicate if they had considered giving up their pet due to COVID-19.

Potential pet-related causes of concern were also listed (e.g., financial difficulties/buying pet food/access to veterinary treatment), and participants were asked to indicate whether any were applicable. Space was given for participants to list any concerns that were not included in the list.

The questions listed regarding the COVID-19 pandemic were all used in previous research conducted during the pandemic [29].

Although all measures were included in the questionnaires sent to participants in the patient group, control questionnaires did not include LMS-QoL or USE-MS questions as these elements are designed to be MS specific.

Therefore, the following sections were asked and completed by the following groups (Table 1):

No incentives were provided for questionnaire completion. Pre-paid, stamped, and addressed envelopes were provided for return of questionnaires. 

### 2.6. Data Processing

Pre-existing guidelines and discussions with the questionnaire developers guided approaches to missing data. The minimum number of questions required to be answered for each questionnaire were as follows:

21 (out of 23) for LAPS.

4 (out of 8) for SPSR.

5 (out of 8) for LMS-QoL.

6 (out of 12) for USE-MS.

Imputation from existing answers was used for missing data if the minimum number of questions had been answered but some were unanswered. Forms with less than the minimum required number of questions completed were excluded.

Questionnaires that were assessed to have been completed erroneously were excluded (e.g., patients completing control questionnaires; the same control completing duplicate questionnaires). Assessment was based on handwriting and answers. 

### 2.7. Statistical Analysis

Descriptive statistics were used for the majority of analysis.

Multiple linear regression was used to explore the contribution of the independent variables of MS status (where applicable), pet ownership, employment status, and living status, to dependent outcome variables SPSR, QoL, and self-efficacy. The number of pets and cat/dog ownership was also considered, as was an interaction term between pet ownership and MS status for SPSR. Each model was controlled for age and sex.

## 3. Results

Of 800 questionnaires posted, 374 questionnaires were returned (198 PwMS and 176 control), representing a 25% response rate calculated for PwMS responses only, as the return of control questionnaires was reliant on the distribution by the patient group. A total of 9 patient questionnaires were excluded based on participants not having received a diagnosis of MS, leaving 189 for further analysis. Overall, 13 control questionnaires were excluded due to duplicate completion, leaving 163 for analysis. 

### 3.1. Demographics

Age was similar between PwMS and controls, but a higher proportion of women responded in the PwMS compared to the control group.

The most common MS subtype was RRMS.

Employment status differed between the two groups, with higher numbers of medically retired individuals in the MS group and higher rates of employment in the control group.

The effects of the pandemic, in terms of time spent at home or out of the home, also differed between the groups, with the MS group having higher numbers of people isolating at home, and the control group being more likely to carry on as normal.

For full details, please see Table 2.

### 3.2. Pet Ownership

Over half of the participants in both the MS and control groups reported owning a pet (PwMS: *n* = 110, 58%, control: *n* = 105, 64%). Two members of the MS group reported spending long periods of time with pets despite not having their own. These people completed the pet-related questions of the questionnaire, but for the purposes of the study, both were recorded as non-pet owners and their answers to any pet-related questions were not included in any data analysis.

Dogs were the most common animal type to be owned in either group (owned by 66% of PwMS and 68% of controls), followed by cats (44% and 48%, respectively). “Other” pets were owned by 10% of PwMS and 19% of controls, and included rabbits, guinea pigs, geckos, horses, birds, and fish.

Further characteristics are presented in Table 3.

Comparing pet owners and non-pet owners showed that in both the MS and the control group, pet owners tended to be younger than non-pet owners. No differences were seen in MS subtypes between pet owners and non-pet owners. 

#### 3.2.1. Pet Ownership and Multiple Sclerosis–Associations with Quality of Life, Satisfaction with Social Roles, and Self-Efficacy 

**Objective 1.** To Explore the Rates of Pet Ownership in People Currently Living with MS and Associations with Quality of Life, Satisfaction with Social Roles and Self-Efficacy Scores in PwMS.

**Hypothesis** **1.** *Pet Ownership Is Associated with Higher Rates of Quality of Life, Self-Efficacy, and Satisfaction with Social Roles in PwMS.*

**Figure 1:**
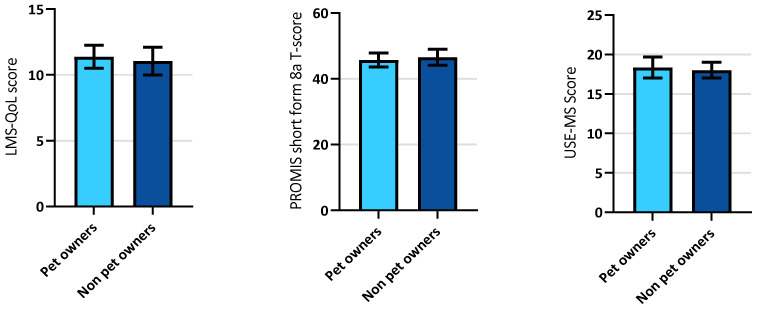
Comparisons of PwMS with and without pets; data displayed as mean +/− 95% confidence intervals. (**A**) Compares Leeds MS-QOL scores between pet owners (*n* = 109; mean 11.38; 95% CI 10.5–12.25) and non-pet owners (*n* = 78; mean 11.05; 95% CI 9.99–12.11). (**B**) Compares PROMIS short-form 8a scores (assessing satisfaction with social roles) between pet owners (*n* = 106; mean = 45.72; 95% CI 43.62–47.81) and non-pet owners (*n* = 78; mean = 46.58; 95% CI 44.12–49.04). (**C**) Compares USE-MS scores between pet owners (*n* = 109; mean = 18.35; 95% CI 17.00–19.69) and non-pet owners (*n* = 79; mean = 18.15; 95% CI 16.71–19.59).

No significant differences were seen in LMS-QOL, SPSR, and USE-MS scores between pet owners and non-pet owners living with MS (see Figure 1).

Using the linear regression models, employment was estimated to impact both QoL (employment reduced the mean QoL score by 2.9 points (standard error 0.79)) and USE-MS (employment increased the mean USE-MS score by 5.7 points (standard error 1.1)). Estimates for other factors, as described in the methods section, were much smaller. 

#### 3.2.2. Pet Attachment

**Objective 2.** To Compare the Levels of Attachment to Pets in PwMS and Controls (People without MS).

**Hypothesis** **2.** *Pet Attachment May Be Higher in PwMS Than in Controls.*

Multiple linear regression was used to determine the effect of having a diagnosis of MS on LAPS score, adjusting for relevant covariates. There was no apparent change in LAPS score seen with having a diagnosis of MS (1.19 (95% CI [−2.48, 4.87]), age (−0.07 per year, 95% CI −0.22, 0.08), or number of pets (−0.02 per pet; 95% CI −0.59, 0.18). Dog ownership was associated with an estimated 8.78 increase (95% CI [3.36, 14.19]) in LAPS and the male gender was associated with an estimated 5.94 decrease (95% CI [−9.89, −2.00]).

#### 3.2.3. Satisfaction with Social Roles in Pet-Owning and Non-Pet-Owning PwMS and Controls 

**Objective 3.** To Compare Levels of Satisfaction with Social Roles in Pet-Owning and Non-Pet-Owning PwMS and People without MS.

**Hypothesis** **3.** *Satisfaction with Social Roles May Be Higher in Pet-Owning Than Non-Pet-Owning PwMS and Controls.*

Overall, rates of satisfaction with social roles (SPSR) were lower in PwMS than in controls (mean (standard deviation) 46.08 (10.87) in PwMS and 56.44 (9.20) in controls; difference between means and 95% CI 10.36 (8.22–12.51)).

**Figure 2:**
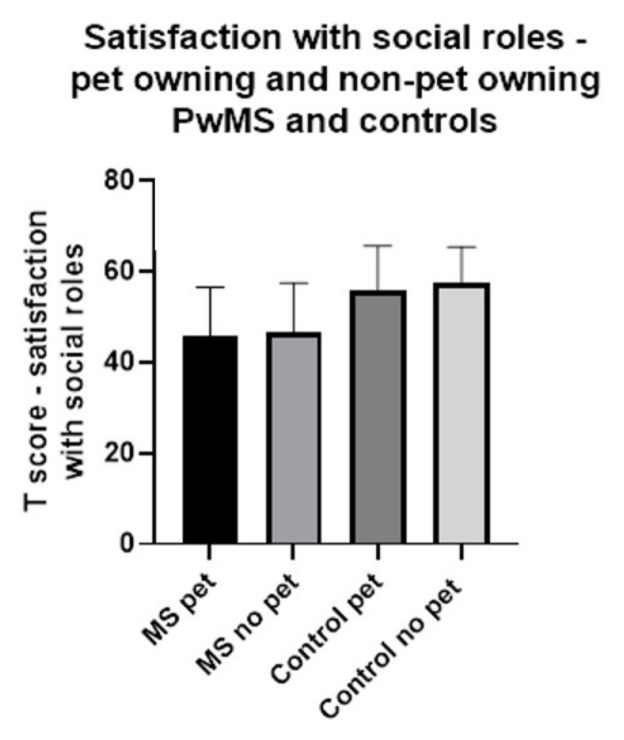
Comparison of satisfaction with social roles between pet owners and non pet owners with and without MS.

There was no apparent difference in SPSR between pet-owning and non-pet-owning PwMS and pet-owning and non-pet-owning controls (see Figure 2).

Using the linear model, the biggest estimated effects on the SPSR score were for: MS status, which reduced the mean SPSR score by 10 points (standard error (SE) = 1.6); employment status, which increased the mean score by 10.6 points (SE = 1.2); and being a pet owner, which reduced the mean score by 3 points (SE = 1.5). Estimates for other factors were much smaller (Table 4).

### 3.3. Pet Ownership during the COVID-19 Pandemic 

**Objective 4.** To Explore Effects of the COVID-19 Outbreak on People’s Perceived Relationships with Their Pets. 

No Matched Hypothesis.

**Figure 3:**
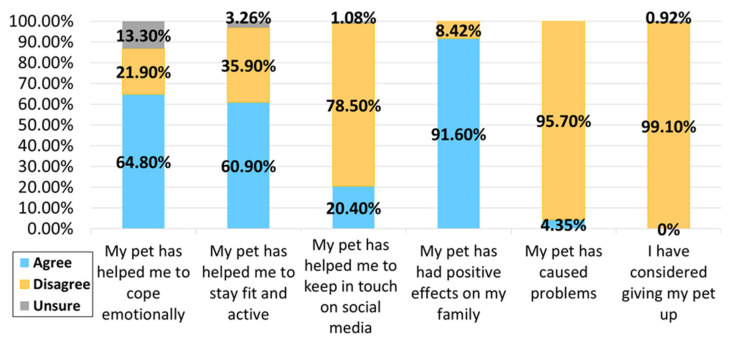
Perceptions of pet-owning PwMS during COVID-19.

**Figure 4:**
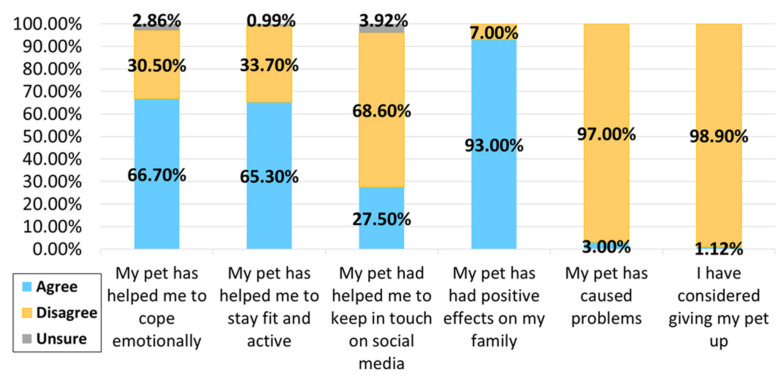
Perceptions of pet-owning people without MS during COVID-19.

In both the control and MS groups, most participants thought that their pet helped them to cope emotionally and stay fit and active, with positive impacts on their family. Very few respondents reported pets causing problems in their family or had considered giving up their pet as a result of the COVID-19 pandemic (see Figure 3 and Figure 4).

#### Pet Ownership-Related Concerns during the COVID-19 Pandemic

In the MS group, 42.7% (*n* = 47) of participants reported having concerns related to pet ownership as a result of the COVID-19 pandemic. In total, these 47 PwMS reported 90 individual concerns. In the control group, this value was slightly lower at just 35.2% (*n* = 37; OR = 1.37; 95% confidence intervals 0.79–2.60), with a total of 65 individual concerns reported. An average of 1.9 concerns were reported per PwMS reporting concerns and 1.8 per person in the control group. The most common concern across both the MS and control groups was access to veterinary care. In the MS group, the next most reported concern was restrictions on exercise, which was ranked third in the control group, along with the impacts of returning to work. The second most common concern reported by the control group was the potential impact of owner illness (see Table 5).

## 4. Discussion

This single-center postal questionnaire study investigated attachment to pets and the associations of pet ownership and attachment on QoL, SPSR, and self-efficacy for people living with MS for the first time. Having MS was not associated with any differences in attachment to pets, but attachment was found to be higher in dog owners and lower in males. Pet ownership and level of attachment were not associated with meaningful differences in QoL, SPSR, or self-efficacy.

The study also explored the perceived roles of pets during the COVID-19 pandemic and found that pets were perceived to have positive effects during this time, particularly as a source of emotional support. However, pet-related concerns were also reported, with access to veterinary treatment representing the greatest worry for participants in both the patient and control groups.

### 4.1. Findings

#### Objectives and Hypotheses

1. To explore the rates of pet ownership and associations with quality of life, satisfaction with social roles, and self-efficacy scores in PwMS/pet ownership is associated with higher rates of quality of life, self-efficacy, and satisfaction with social roles in PwMS

Finding: Although several studies have found pet ownership to improve social participation and loneliness, provide emotional support, promote physical exercise, and in some cases ameliorate chronic pain, a factor known to be associated with a reduced QoL [11,12,15], the study found no difference in QoL, SPSR, and self-efficacy between pet owners and non-pet owners living with MS on initial analysis. Introducing a linear model, with independent variables of age and sex, MS status, pet ownership, living alone, and employment status, showed that:

- MS was associated with a reduction in satisfaction with social roles.
- Employment was associated with increased satisfaction with social roles.
- Employment was associated with improved quality of life.
- Employment was associated with increased self-efficacy.
- Pet ownership was associated with a reduction in satisfaction with social roles.

This is in keeping with established findings that doing a job of work (that one enjoys) is psychosocially beneficial [38,39] and that MS is associated with lower satisfaction with social roles [22]. 

No associations were seen between LAPS score and QoL, self-efficacy, and SPSR in PwMS, which is in keeping with previous work reporting no significant relationship between attachment to pets, perceived stress, and life satisfaction [40]. Despite this, evidence of an association between attachment and health-related benefits in cat owners, and attachment and emotional support in dog owners implies that, while attachment may not be linked to improved overall QoL, self-efficacy, and SPSR, it may be associated with other important benefits that could vary with pet species [41,42].

2. To compare the levels of attachment to pets in PwMS and people without MS/pet attachment may be higher in PwMS than in controls.

Finding: Our findings did not support an effect of MS on the level of attachment to pets. In keeping with previous work, females appeared to experience greater levels of attachment than males [40,43]. Dog ownership also appeared to be associated with a greater LAPS score, whilst the effect of cat ownership was unclear. Increased LAPS scores as a result of dog ownership have been reported previously (although not in recent COVID-19-related work [29]) and could be attributed to the greater amounts of time spent with dogs compared to other animals due to an increased requirement for more individual care including training and exercise [42,43,44].

3. To compare levels of satisfaction with social roles in pet-owning and non-pet-owning PwMS and people without MS/satisfaction with social roles may be higher in pet-owning than non-pet-owning PwMS and controls

Finding: The finding of a reduction in satisfaction with social roles (SPSR) being associated with pet ownership was contrary to our hypothesis. It is possible that, particularly during the time of a pandemic, during a period of heightened stress for many people, pet ownership added an extra “burden” of work, as the PROMIS Short Form 8a does ask specifically about being satisfied with one’s “ability to meet the needs of those who depend on me”, which may be particularly important in view of the high rates of concerns about pet ownership raised by the respondents to our questionnaires.

4. To explore the effects of the COVID-19 outbreak on people’s perceived relationships with their pets (how the participants thought their pets helped them emotionally and physically and whether they had any concerns about being a pet owner during the COVID-19 pandemic)/no matched hypothesis.

The majority of pet owners in both MS and control groups reported that they felt that their pets had beneficial effects on their health and wellbeing during the COVID-19 pandemic. Pets appear to be an important source of emotional support, whilst also promoting physical exercise, enabling owners to keep fit and active, a benefit that may be felt particularly amongst dog owners, who have been found previously to participate in more mild to moderate exercise than other pet owners [29,44,45]. This is important in the context of the COVID-19 pandemic, which has resulted in poor mental health across all groups, as well as restrictions on normal activities [25]. However, for PwMS, a group known to experience high levels of poor mental health, isolation, and reduced exercise, under normal life conditions, an additional source of emotional support in the form of a companion may be particularly beneficial [2,46]. The majority (>90%) of respondents in both the patient and control groups felt that their pets had been beneficial for the whole family, during a period that posed a threat to interpersonal relationships due to high levels of stress and social disruption [47].

The body of literature regarding pet ownership and COVID-19 is growing. There are generally high levels of agreement that pet ownership can have beneficial effects to mitigate some of the negative impacts of lockdown, such as low mood, loneliness, and isolation [29,45,48,49]. People with MS and other disabilities are more likely to experience these phenomena without being in a pandemic-related lockdown [50,51,52] and so may be particularly likely to benefit from pet ownership. 

Overall, almost no participants reported that they had considered giving up their pet due to COVID-19, indicating that concerns around pet ownership were not sufficient to cause participants to want to give up their animal. However, it has been recognized that during the pandemic, there have been high rates of adoption or purchasing of pets; as people return to work, and to the “new normal”, it will be important to see if this situation changes [53].

Comparisons of the concerns reported by the MS and control group showed similar rates of concern in both groups but at lower rates than those reported in some previous studies [29,30]. This could be the result of the relatively small sample size used or differences in sample populations, for example, due to differing methods of participant recruitment (online versus postal; open to any respondents versus selected participants). The particular areas of concern were similar to those shown in previous studies, around access to veterinary treatment [29] and effects on animal exercise, activity and otherwise meeting the needs of pets [30]. One study has suggested that pet ownership during COVID was associated with lower life satisfaction, perhaps because of some of these areas of concern [54], but the responses from our questionnaire were overall more positive than this, with lower rates of concerns and considerations regarding potentially giving up their pets among our respondents. Numbers of people with concerns and numbers of concerns per concerned person were not different between PwMS and controls, suggesting that having MS did not make people be more likely to have concerns about the effects of COVID-19 on their pet, or to have a greater number of concerns. However, while access to veterinary treatment was the most reported concern by both groups, access to food and restrictions on exercise were ranked higher by the patient group than the control group. These differences could be contributed to by a greater level of concern about catching COVID-19 and its potential effects amongst PwMS, especially as the most reported MS subtype was RRMS, the subtype most associated with use of disease-modifying therapies (DMTs) that can affect the immune system [28,55,56]. It is also relevant that there was a higher proportion of shielding among PwMS responding to our questionnaire than in the controls, which may have affected the ability to access veterinary treatment, shopping, and exercise. Additionally, concerns about returning to work were ranked higher by the control than the MS group, potentially due to the lower rates of employment among the patient group compared to the control group.

Concerns related to the impact of owner illness on pet welfare were widely reported in both groups. It is an important area to address, especially as a study in the US reported that 10% of pet owners might delay or avoid testing for COVID-19, and over 10% might delay or avoid treatment, due to concerns about the welfare of their pet, with the lack of adequate planning for pet care being cited as a key contributing factor by many pet owners [57]. This could potentially provide a rationale for the promotion of suitable planning for pets in cases of illness, especially by people with long-term conditions who, under normal circumstances, may be more likely to experience sudden difficulties with caring for their pet.

### 4.2. Limitations

A key limitation of the study was the relatively small sample size and the low response rate without power calculations, which meant that, although we were able to explore relationships, we were unable to comment on their significance. Additionally, recruitment of the control group was dependent on the patient group, which may have led to a biased representation as the friends and family of the patient group may be more likely to have similar opinions or experiences to the patient group. 

Our questionnaire was designed to be brief and simple to complete, but this meant that we did not collect some data that could have been relevant to the study. We only asked about MS as a diagnosis and no other illnesses, conditions, or disabilities. We only asked about current pet ownership and did not analyze for factors, such as length of pet ownership, current health status of pets, or previous pet ownership and subsequent loss. Furthermore, there are many other variables that could mediate the relationship between pet ownership and outcome (e.g., number of pets/perceived friendliness of pets/other sources of social support, etc.), which may have contributed to our findings but which were not recorded [11,58].

The risk of bias was likely even greater due to the COVID-19 pandemic, which may have limited the number of people available to ask to complete the survey, leading to family members, carers, or at least members of the same household being more likely to complete the questionnaire. If this is indeed the case, then it is possible that the controls’ relationships with a PwMS could have influenced some of their responses, as a chronic illness, such as MS, affects all family members, not just the individual patient [59]. The patient and control group had some important differences, including a higher preponderance of females in the patient than the control group (which would fit with the female preponderance in this condition). PwMS were more likely to be medically retired or work from home part-time, whereas controls were more likely to be working out of the house full time. COVID-19 also impacts on patients and controls differently, with controls being more likely to report carrying on as normal during the pandemic, and patients more likely to report isolating at home for longer periods. As a result, in the future, further research should involve more stringent recruitment of a representative control group to improve the generalizability of the results. Although we made every effort to include only essential questions, and instructed respondents to ask for help or to take breaks if required, the questionnaire was still quite lengthy, at eight pages long, and for people struggling with fatigue, cognitive impairment, difficulties with reading or writing, or poor concentration for whatever reason, this may have been overwhelming or simply not possible. There were, perhaps, some steps that we could have taken to increase the response rate, such as posting out reminders [60], using an incentive, colored ink, or recorded delivery [61], but finances were limited for this study. However, these would be important factors to consider for future work.

Answers to the LMS-QoL, USE-MS and PROMIS short form 8a (SPSR) may also have been influenced by the COVID-19 pandemic, with answers to questions, such as ‘I have felt lonely’ (LMS-QoL), ‘I find that the things I do in the day make me feel happy and satisfied’ (USE-MS), and ‘I am satisfied with my ability to work’ (PROMIS SF 8a), potentially being impacted by the increased isolation and poor mental health associated with the pandemic [25,27]. Additionally, when analyzing the LAPS results, we only controlled for a small number of factors known to influence the score. Therefore, future research should aim to control for a wider range of factors including marital status and potentially the level of pet caring responsibility [31].

While we chose to use a postal questionnaire to try to ensure accessibility and allow respondents to take as much time as required to complete it, we recognize that the response rates were low, and could perhaps have been improved by approaches used in other postal surveys (e.g., personalization, follow-up contacts, incentives, etc.) [60,61].

The complexity of human–animal interactions and relationships, and their effects on psychosocial wellbeing arguably may not lend themselves to such quantitative work, and future work could benefit from including qualitative research, which could better explore some of these intricacies, including the effects of living with chronic illnesses and the contribution of pets to the day-to-day management of MS. It will also be important to embed patient and public involvement in such future work to ensure that any outcomes studied are relevant to the experience of living with MS.

## 5. Conclusions

This study has added to the current body of knowledge on human–animal interactions. Pet ownership was not found to affect QoL, self-efficacy, or SPSR in PwMS. However, pet ownership has previously been found to positively affect participation and mental health, as well as chronic pain, and participants in this study self-reported benefits of pet ownership. Therefore, further research exploring the impacts of pet ownership as a single facet of life is required to better understand its contribution to QoL, self-efficacy, and SPSR. The cross-sectional nature of this work also precluded us from investigating temporal relationships between the different factors (e.g., whether a PwMS had a lower self-efficacy score before becoming a pet owner). Relatively few studies have looked at such relationships, although there is evidence that becoming a dog owner can reduce loneliness levels [62].

Concerns around the impact of owner illness on pet welfare, and evidence from previous studies that some people would delay or avoid treatment because of this, highlight the importance of effective pet care plans (i.e., plans for temporary care arrangements for companion animals if an owner becomes unwell and/or needs hospital admission) as well as the need for healthcare professionals to consider how pet ownership may affect treatment-related decisions [57]. This is particularly important for people living with long-term conditions, such as MS, who may be at an increased risk of sudden illness.

## Figures and Tables

**Table 1:** Information collected from participant groups.

	Pet-Owning PwMS	Non-Pet-Owning Pwms	Pet-Owning Controls	Non-Pet-Owning Controls
Age, gender, employment status	✓	✓	✓	✓
MS subtype	✓	✓		
Pet ownership (yes/no)	✓	✓	✓	✓
Details of current pets (type and number)	✓		✓	
Attachment to pets (LAPS)	✓		✓	
Satisfaction with social roles (SPSR)	✓	✓	✓	✓
Quality of life (QoL/LMS-QoL)	✓	✓		
Self-efficacy (USE-MS)	✓	✓		
Pets during COVID-19	✓		✓	

**Table 2:** Characteristics of participants. OR: odds ratio. CI: confidence interval (95%).

	PwMS (*n* = 189)	Control Group (*n* = 163)	Comparison
**Age** (mean, standard deviation), years	54, 12	53, 16	Difference in means with 95% CI −1 (−4 to +2)
**Gender** male: female: not answered N (%)	44: 143: 2 (23: 76: 1)	77: 82: 4 (47: 50: 2)	OR 0.33; 95% CI 0.21–0.52
**Multiple sclerosis subtype N** (%) PPMS		n/a	n/a
	15 (8)		
PRMS	5 (3)		
RRMS	109 (58)		
SPMS	44 (23)		
Unsure	11 (6)		
Not answered	5 (3)		
**Employment status** N (%) Retired	41 (22)	48 (29)	OR and 95% CI: 0.66 (0.40–1.08)
Medically retired	42 (22)	5 (3)	**9.03 (3.5–21.5)**
Full time out of the house	37 (20)	57 (35)	**0.45 (0.28–0.73)**
Full time working from home	11 (6)	11 (7)	0.85 (0.34–1.93)
Part time out of the house	25 (13)	26 (16)	0.80 (0.44–1.48)
Part time working from home	17 (9)	5 (3)	**3.1 (1.15–7.86)**
Unemployed	14 (7)	7 (4)	1.78 (0.70–4.61)
Other	1 (1)	4 (2)	0.21 (0.02–1.30)
Not answered	1 (1)	0 (0)	
**Pet owner** N (%) Yes	110 (58)	105 (64)	OR 0.77; 95% CI 0.49–1.12
No	79 (42)	58 (36)	
**Effects of pandemic** N (%) Carried on as normal	29 (15)	62 (38)	OR and 95% CI: 0.30 (0.18–0.49)
Socially distanced but left house regularly	82 (43)	77 (47)	0.86 (0.56–1.30)
Isolated at home for less than 7 days	8 (4)	4 (2)	1.76 (0.53–5.32)
Isolated at home for 8–14 days	5 (3)	4 (2)	1.08 (0.32–3.57)
Isolated at home for more than 14 days	62 (32)	16 (10)	**4.49 (2.50–8.10)**
Not answered	3 (2)	0 (0)	

**Table 3:** Characteristics of pet owners and non-pet owners in the patient and control groups.

	PwMS–Pet Owners (*n* = 110)	PwMS–Non-Pet Owners (*n* = 79)	Comparison (PwMS)	Control Group–Pet Owners (*n* = 105)	Control Group–Non-Pet Owners (*n* = 58)	Comparison (Control Group)
**Age** mean, SD	52.0, 11.2	56.0, 12.2	Difference in means with 95% CI 4.0 (0.54–7.36)	49.6, 13.4	57.9, 17.8	Difference in means with 95% CI 8.3 (3.4–13.2)
**Gender** M: F: not answered N (%)	23: 85: 2 (21: 77: 2)	21: 58: 0 (27: 73: 0)	OR 0.75; 95% CI 0.38–1.47	46: 56: 3 (44: 53: 3)	31: 26: 1 (53: 45: 1)	OR 0.69; 95% CI 0.36–1.33
**MS subtype** N (%) PPMS	5 (5)	10 (13)	OR with 95% CI 0.34 (0.12–0.96)	n/a	n/a	n/a
PRMS	4 (4)	1 (1)	3.02 (0.48–37.38)			
RRMS	64 (58)	45 (57)	1.17 (0.61–2.03)			
SPMS	26 (24)	18 (23)	1.08 (0.56–2.22)			
Unsure	7 (6)	4 (5)	1.31 (0.40–4.11			

**Table 4:** Summary table of questionnaire results within participant groups.

	Pet-Owning PwMS	Non-Pet-Owning PwMS	Pet-Owning Controls	Non-Pet-Owning Controls
Attachment to pets (LAPS score) mean and 95% CI	49.0 (46.5–51.6)	n/a	46.3 (43.4–49.1)	n/a
Satisfaction with social roles (SPSR) T score mean and 95% CI	45.7 (43.6–47.8)	46.6 (44.1–49.0)	55.8 (53.9–57.8)	57.5 (55.5–59.6)
Quality of life LMS-QOL mean score with 95% CI	11.4 (10.5–12.3)	11.1 (10.0–12.1)	n/a	n/a
Self-efficacy USE-MS mean score with 95% CI	18.4 (17.0–19.7)	18.2 (16.7–19.6)	n/a	n/a

**Table 5:** Pet ownership-related concerns reported by PwMS and controls during the COVID-19 pandemic.

Nature of Concern	% (Number) Respondents in MS Group Reporting Concern (*n* = 110)	% (Number) Respondents in Control Group Reporting Concern (*n* = 105)
**Access to veterinary care**	28 (31)	18 (19)
**Exercise restrictions**	14 (15)	8 (8)
**Access to food and supplies**	11 (12)	6 (6)
**Impact of illness**	10 (11)	12 (13)
**Financial concerns**	8 (9)	6 (6)
**Return to work**	5 (6)	8 (8)
**Changes to routine**	4 (4)	2 (2)
**COVID infection from pets**	1 (1)	2 (2)
**Other**	1 (1)	1 (1)

## References

[1] Rommer P.S., Eichstädt K., Ellenberger D., Flachenecker P., Friede T., Haas J., Kleinschnitz C., Pöhlau D., Rienhoff O., Stahmann A. (2019). Symptomatology and symptomatic treatment in multiple sclerosis: Results from a nationwide MS registry. Mult. Scler. J..

[2] Motl R.W., Snook E.M., McAuley E., Gliottoni R.C. (2006). Symptoms, self-efficacy, and physical activity among individuals with multiple sclerosis. Res. Nurs. Health.

[3] Compston A., Coles A. (2008). Multiple sclerosis. Lancet.

[4] Barin L., Salmen A., Disanto G., Babačić H., Calabrese P., Chan A., Kamm C.P., Kesselring J., Kuhle J., Gobbi C. (2018). The disease burden of Multiple Sclerosis from the individual and population perspective: Which symptoms matter most?. Mult. Scler. Relat. Disord..

[5] Janardhan V., Bakshi R. (2000). Quality of life and its relationship to brain lesions and atrophy on magnetic resonance images in 60 patients with multiple sclerosis. Arch. Neurol..

[6] Hermann B.P., Vickrey B., Hays R.D., Cramer J., Devinsky O., Meador K., Perrine K., Myers L.W., Ellison G.W. (1996). A comparison of health-related quality of life in patients with epilepsy, diabetes and multiple sclerosis. Epilepsy Res..

[7] Murphy R., O’Donoghue S., Counihan T., McDonald C., Calabresi P., Ahmed M.A., Kaplin A., Hallahan B. (2017). Neuropsychiatric syndromes of multiple sclerosis. J. Neurol. Neurosurg. Psychiatry.

[8] Kwiatkowski A., Marissal J.-P., Pouyfaucon M., Vermersch P., Hautecoeur P., Dervaux B. (2014). Social participation in patients with multiple sclerosis: Correlations between disability and economic burden. BMC Neurol..

[9] Allen K., Blascovich J., Mendes W.B. (2002). Cardiovascular Reactivity and the Presence of Pets, Friends, and Spouses: The Truth About Cats and Dogs. Psychosom. Med..

[10] Irani S., Mahler C., Goetzmann L., Russi E., Boehler A. (2006). Lung Transplant Recipients Holding Companion Animals: Impact on Physical Health and Quality of Life. Arab. Archaeol. Epigr..

[11] Brooks H., Rushton K., Walker S., Lovell K., Rogers A. (2016). Ontological security and connectivity provided by pets: A study in the self-management of the everyday lives of people diagnosed with a long-term mental health condition. BMC Psychiatry.

[12] Brooks H.L., Rogers A., Kapadia D., Pilgrim J., Reeves D., Vassilev I. (2012). Creature comforts: Personal communities, pets and the work of managing a long-term condition. Chronic Illn..

[13] Wisdom J.P., Saedi G.A., Green C.A. (2009). Another breed of “service” animals: STARS study findings about pet ownership and recovery from serious mental illness. Am. J. Orthopsychiatry.

[14] Wells D.L. (2009). Associations Between Pet Ownership and Self-Reported Health Status in People Suffering from Chronic Fatigue Syndrome. J. Altern. Complement. Med..

[15] Ryan S., Ziebland S. (2015). On interviewing people with pets: Reflections from qualitative research on people with long-term conditions. Sociol. Health Illn..

[16] Bandura A. (1977). Self-efficacy: Toward a unifying theory of behavioral change. Psychol. Rev..

[17] Bonsaken T., Lerdal A., Fagermoan M.S. (2012). Factors associated with self-efficacy in persons with chronic illness. Scand. J. Psychol..

[18] Jongen P.J., Heerings M., Ruimschotel R., Hussaarts A., Duyverman L., Van Der Zande A., Valkenburg-Vissers J., Van Droffelaar M., Lemmens W., Donders R. (2016). Intensive social cognitive treatment (can do treatment) with participation of support partners in persons with relapsing remitting multiple sclerosis: Observation of improved self-efficacy, quality of life, anxiety and depression 1 year later. BMC Res. Notes.

[19] Jongen P.J., Ruimschotel R., Heerings M., Hussaarts A., Duyverman L., Van Der Zande A., Valkenburg-Vissers J., Wolper H., Van Droffelaar M., Lemmens W. (2014). Improved self-efficacy in persons with relapsing remitting multiple sclerosis after an intensive social cognitive wellness program with participation of support partners: A 6-months observational study. Health Qual. Life Outcomes.

[20] De Guzman A.B., Cucueco D.S., Cuenco I.B.V., Cunanan N.G.C., Dabandan R.T., Dacanay E.J.E. (2009). Petmanship: Understanding Elderly Filipinos’ Self-Perceived Health and Self-Esteem Captured from their Lived Experiences with Pet Companions. Educ. Gerontol..

[21] Battalio S.L., Jensen M.P., Molton I.R. (2019). Secondary health conditions and social role satisfaction in adults with long-term physical disability. Health Psychol..

[22] Amtmann D., Bamer A.M., Kim J., Chung H., Salem R. (2018). People with multiple sclerosis report significantly worse symptoms and health related quality of life than the US general population as measured by PROMIS and NeuroQoL outcome measures. Disabil. Health J..

[23] Sturgeon J.A., Dixon E.A., Darnall B.D., Mackey S.C. (2015). Contributions of physical function and satisfaction with social roles to emotional distress in chronic pain: A Collaborative Health Outcomes Information Registry (CHOIR) study. Pain.

[24] Krause-Parello C.A. (2012). Pet Ownership and Older Women: The Relationships Among Loneliness, Pet Attachment Support, Human Social Support, and Depressed Mood. Geriatr. Nurs..

[25] Jia R., Ayling K., Chalder T., Massey A., Broadbent E., Coupland C., Vedhara K. (2020). Mental health in the UK during the COVID-19 pandemic: Cross-sectional analyses from a community cohort study. BMJ Open.

[26] Costabile T., Carotenuto A., Lavorgna L., Borriello G., Moiola L., Inglese M., Petruzzo M., Trojsi F., Ianniello A., Nozzolillo A. (2021). COVID-19 pandemic and mental distress in multiple sclerosis: Implications for clinical management. Eur. J. Neurol..

[27] Motolese F., Rossi M., Albergo G., Stelitano D., Villanova M., Di Lazzaro V., Capone F. (2020). The Psychological Impact of COVID-19 Pandemic on People with Multiple Sclerosis. Front. Neurol..

[28] Shaygannejad V., Afshari-Safavi A., Hatef B. (2020). Assessment of mental health, knowledge, and attitude of patients with multiple sclerosis and neuromyelitis optica spectrum disorder in response to 2019 novel coronavirus. Neurol. Sci..

[29] Ratschen E., Shoesmith E., Shahab L., Silva K., Kale D., Toner P., Reeve C., Mills D.S. (2020). Human-animal relationships and interactions during the Covid-19 lockdown phase in the UK: Investigating links with mental health and loneliness. PLoS ONE.

[30] Applebaum J.W., Tomlinson C.A., Matijczak A., McDonald S.E., Zsembik B.A. (2020). The Concerns, Difficulties, and Stressors of Caring for Pets during COVID-19: Results from a Large Survey of U.S. Pet Owners. Animals.

[31] Johnson T.P., Garrity T.F., Stallones L. (1992). Psychometric Evaluation of the Lexington Attachment to Pets Scale (Laps). Anthrozoös.

[32] Cella D., Choi S.W., Condon D.M., Schalet B., Hays R.D., Rothrock N.E., Yount S., Cook K.F., Gershon R.C., Amtmann D. (2019). PROMIS® Adult Health Profiles: Efficient Short-Form Measures of Seven Health Domains. Value Health.

[33] Segawa E., Schalet B., Cella D. (2020). A comparison of computer adaptive tests (CATs) and short forms in terms of accuracy and number of items administrated using PROMIS profile. Qual. Life Res..

[34] Ford H.L., Gerry E., Tennant A., Whalley D., Haigh R., Johnson M.H. (2001). Developing a disease-specific quality of life measure for people with multiple sclerosis. Clin. Rehabil..

[35] Nagaraj K., Taly A.B., Gupta A., Prasad C., Christopher R. (2013). Prevalence of fatigue in patients with multiple sclerosis and its effect on the quality of life. J. Neurosci. Rural. Pract..

[36] Ensari I., Motl R.W., McAuley E. (2015). Structural and construct validity of the Leeds Multiple Sclerosis Quality of Life scale. Qual. Life Res..

[37] Young C., Mills R., Woolmore J., Hawkins C., Tennant A., Young C. (2012). The unidimensional self-efficacy scale for MS (USE-MS): Developing a patient based and patient reported outcome. Mult. Scler. J..

[38] Modini M., Joyce S., Mykletun A., Christensen H., Bryant R.A., Mitchell P.B., Harvey S.B. (2016). The mental health benefits of employment: Results of a systematic meta-review. Australas. Psychiatry.

[39] Zhao Y., Zhou Z., Fan X., Nawaz R., Zhao D., Xu T., Su M., Cao D., Shen C., Lai S. (2021). Comparison of inequity in health-related quality of life among unemployed and employed individuals in China. BMC Public Health.

[40] Le Roux M.C., Wright S. (2020). The Relationship Between Pet Attachment, Life Satisfaction, and Perceived Stress: Results from a South African Online Survey. Anthrozoös.

[41] Dinis F.A., Martins T.L.F. (2016). Does cat attachment have an effect on human health? A comparison between owners and volunteers. Pet Behav. Sci..

[42] Joseph N., Chandramohan A.K., D’Souza A.L., Basavanna S.C., Hariram S., Nayak A.H. (2019). Assessment of pet attachment and its relationship with stress and social support among residents in Mangalore city of south India. J. Vet. Behav..

[43] Smolkovic I., Fajfar M., Mlinaric V. (2012). Attachment to pets and interpersonal relationships: Can a four-legged friend replace a two-legged one?. J. Eur. Psychol. Stud..

[44] Mein G., Grant R. (2018). A cross-sectional exploratory analysis between pet ownership, sleep, exercise, health and neighbourhood perceptions: The Whitehall II cohort study. BMC Geriatr..

[45] Oliva J.L., Johnston K.L. (2020). Puppy love in the time of Corona: Dog ownership protects against loneliness for those living alone during the COVID-19 lockdown. Int. J. Soc. Psychiatry.

[46] Boeschoten R.E., Braamse A.M., Beekman A.T., Cuijpers P., van Oppen P., Dekker J., Uitdehaag B.M. (2017). Prevalence of depression and anxiety in Multiple Sclerosis: A systematic review and meta-analysis. J. Neurol. Sci..

[47] Prime H., Wade M., Browne D.T. (2020). Risk and resilience in family well-being during the COVID-19 pandemic. Am. Psychol..

[48] Owczarczak-Garstecka S., Graham T., Archer D., Westgarth C. (2021). Dog Walking before and during the COVID-19 Pandemic Lockdown: Experiences of UK Dog Owners. Int. J. Environ. Res. Public Health.

[49] Gasteiger N., Vedhara K., Massey A., Jia R., Ayling K., Chalder T., Coupland C., Broadbent E. (2021). Depression, anxiety and stress during the COVID-19 pandemic: Results from a New Zealand cohort study on mental well-being. BMJ Open.

[50] Burholt V., Windle G., Morgan D.J. (2016). Cfas Wales on behalf of the CFAS Wales team A Social Model of Loneliness: The Roles of Disability, Social Resources, and Cognitive Impairment. Gerontologist.

[51] Freeman J., Gorst T., Gunn H., Robens S. (2020). “A non-person to the rest of the world”: Experiences of social isolation amongst severely impaired people with multiple sclerosis. Disabil. Rehabil..

[52] Beal C.C., Stuifbergen A. (2007). Loneliness in Women with Multiple Sclerosis. Rehabil. Nurs..

[53] Ho J., Hussain S., Sparagano O. (2021). Did the COVID-19 Pandemic Spark a Public Interest in Pet Adoption?. Front. Vet. Sci..

[54] Phillipou A., Tan E., Toh W., Van Rheenen T., Meyer D., Neill E., Sumner P., Rossell S. (2021). Pet ownership and mental health during COVID -19 lockdown. Aust. Vet. J..

[55] Radulovic L., Erakovic J., Roganovic M. (2020). Attitudes of patients with relapsing-remitting form of multiple sclerosis using disease-modifying drugs in Montenegro regarding COVID-19 pandemic. Mult. Scler. Relat. Disord..

[56] Kalron A., Dolev M., Greenberg-Abrahami M., Menascu S., Frid L., Avrech-Shezifi S., Harari G., Magalashvili D., Achiron A. (2021). Physical activity behavior in people with multiple sclerosis during the COVID-19 pandemic in Israel: Results of an online survey. Mult. Scler. Relat. Disord..

[57] Applebaum J.W., Adams B.L., Eliasson M.N., Zsembik B.A., McDonald S.E. (2020). How pets factor into healthcare deci-sions for COVID-19: A One Health perspective. One Health.

[58] Brooks H.L., Rushton K., Lovell K., Bee P., Walker L., Grant L., Rogers A. (2018). The power of support from companion animals for people living with mental health problems: A systematic review and narrative synthesis of the evidence. BMC Psychiatry.

[59] Uccelli M.M. (2014). The impact of multiple sclerosis on family members: A review of the literature. Neurodegener. Dis. Manag..

[60] Nakash R.A., Hutton J.L., Jørstad-Stein E.C., Gates S., Lamb S. (2006). Maximising response to postal questionnaires—A systematic review of randomised trials in health research. BMC Med Res. Methodol..

[61] Edwards P., Roberts I., Clarke M., DiGuiseppi C., Pratap S., Wentz R., Kwan I. (2002). Increasing response rates to postal questionnaires: Systematic review. BMJ.

[62] Powell L., Edwards K.M., McGreevy P., Bauman A., Podberscek A., Neilly B., Sherrington C., Stamatakis E. (2019). Companion dog acquisition and mental well-being: A community-based three-arm controlled study. BMC Public Health.

